# Comparative transcriptomic analyses of two sugarcane *Saccharum* L. cultivars differing in drought tolerance

**DOI:** 10.3389/fpls.2023.1243664

**Published:** 2023-10-11

**Authors:** Haibi Li, Yiyun Gui, Kai Zhu, Jinju Wei, Ronghua Zhang, Rongzhong Yang, Liqiu Tang, Hui Zhou, Xihui Liu

**Affiliations:** ^1^ Sugarcane Research Center of Chinese Academy of Agricultural Sciences, Nanning, China; ^2^ Guangxi Key Laboratory of Sugarcane Genetic Improvement, Guangxi Academy of Agricultural Sciences, Nanning, China; ^3^ Guangxi South Subtropical Agricultural Science Research Institute, Guangxi Academy of Agricultural Sciences, Chongzuo, China

**Keywords:** sugarcane, drought, transcriptome, variety, metabolic pathways, photosynthesis

## Abstract

Sugarcane (*Saccharum* spp.) is an important cash crop, and drought is an important factors limiting its yield. To study the drought resistance mechanism of sugarcane, the transcriptomes of two sugarcane varieties with different levels of drought resistance were compared under different water shortage levels. The results showed that the transcriptomes of the two varieties were significantly different. The differentially expressed genes were enriched in starch and sucrose metabolism, linoleic acid metabolism, glycolysis/gluconeogenesis, and glyoxylate and dicarboxylate metabolic pathways. Unique trend genes of the variety with strong drought resistance (F172) were significantly enriched in photosynthesis, mitogen-activated protein kinases signaling pathway, biosynthesis of various plant secondary metabolites, and cyanoamino acid metabolism pathways. Weighted correlation network analysis indicated that the blue4 and plum1 modules correlated with drought conditions, whereas the tan and salmon4 modules correlated with variety. The unique trend genes expressed in F172 and mapped to the blue4 module were enriched in photosynthesis, purine metabolism, starch and sucrose metabolism, beta-alanine metabolism, photosynthesis-antenna proteins, and plant hormone signal transduction pathways. The expression of genes involved in the photosynthesis-antenna protein and photosynthesis pathways decreased in response to water deficit, indicating that reducing photosynthesis might be a means for sugarcane to respond to drought stress. The results of this study provide insights into drought resistance mechanisms in plants, and the related genes and metabolic pathways identified may be helpful for sugarcane breeding in the future.

## Introduction

Drought stress is a major factor limiting global agricultural production, and the development of drought-resistant crop varieties is of great significance in modern agriculture (Ozturk et al., 2021; Conti et al., 2023). The cultivation of drought-resistant crop varieties requires an understanding of the damage inflicted by drought and the mechanisms of crop drought resistance. Drought stress can affect the basic physiological activities of plants, such as enzymatic function, osmotic pressure, and energy supply, and inhibit mitosis and normal cell metabolism (Tardieu et al., 2018). In response, plants have evolved a series of mechanisms to overcome drought stress or drought-stress conditions, such as closing the stomata to reduce water loss from transpiration, regulating osmotic pressure, altering the expression of numerous genes, adjusting photosynthesis, modulating abscisic acid, and pigment levels, and altering sugar metabolism (Agurla et al., 2018; Conti et al., 2023).

Sugarcane (*Saccharum* spp.) is an economically important crop that can be used as food, feed, and fuel, and has strict water requirements for cultivation (Meena et al., 2020; Dinesh Babu et al., 2022). To adapt to water scarcity, sugarcane has evolved drought resistance mechanisms involving morphological and physiological responses, such as abscisic acid accumulation, ROS scavenging and antioxidant activity, lipid peroxidation and altered expression of certain genes (Ferreira et al., 2017). As the basis of life function, gene expression and its products play a central role in drought resistance of crops. For example, upon exposure to drought stress, dirigent proteins exhibit significant transcriptional responses and improve physiological and biochemical indices (Gentile et al., 2015). Studies have shown that miRNA-mediated post-transcriptional regulation plays an important role in drought resistance in sugarcane, particularly in regulating the production of transcription factors, transporters, senescence-related proteins, and proteins associated with flower development (Ferreira et al., 2017). The ScDREB2B-1 gene cloned from the *Saccharum* spp. hybrid ROC22 responds to drought stress by regulating the abscisic acid signaling pathway, ROS levels, and stress-related gene expression (Li et al., 2022). In addition, the expression of ShCBSD-PB1-5A and ShCBSD-PB1-7A-1 significantly decreased, whereas that of SsCBSDCBS-5A distinctly increased in ROC22 cells in response to drought stress (Gentile et al., 2013). Most of these studies have focused on one aspect of gene expression; however, to gain a comprehensive understanding of gene expression under water-deficit conditions, it is necessary to focus on the expression of all genes, and the rise of sequencing and transcriptomic technologies provides a technical means to solve this problem.

Since the publication of the whole-genome sequence of *Arabidopsis thaliana* in December 2000, research on crop plants has undergone significant advances, such as genome sequencing, and decoding of gene expression and function during development, and during the response to various environmental stimuli (Chen et al., 2022). With the development of sequencing and omics technologies, transcriptome analysis has been widely used to study the relationships between various factors and drought resistance in sugarcane, including those among varieties (Meena et al., 2020). A previous study showed that drought conditions can cause changes in the expression of many sugarcane genes. A total of 3,389 genes have been identified in wild sugarcane exposed to drought stress, including 1,772 upregulated and 1,617 downregulated genes (Belesini et al., 2017). Leaf transcriptomic analysis has shown that the expression of genes related to water retention, antioxidant secondary metabolite biosynthesis, oxidation, and osmotic stress responses is higher in the drought-tolerant sugarcane genotype, while the sensitive genotype has a higher number of downregulated genes, which include those involved in photosynthesis, carbon fixation, and the Calvin cycle (Nawae et al., 2020). A similar study showed that the drought-tolerant genotype Co-06022 expressed more genes than the drought-susceptible genotype Co-8021 under different degrees of drought stress. However, more genes are expressed in sensitive genotypes during the recovery period (Selvi et al., 2020). The results of these studies indicated that the relationship between drought resistance and sugarcane varieties is closely related gene expression at the transcriptome level under drought conditions. In addition, different parts of the sugarcane plant respond differently to drought stress. Fewer genes are upregulated and downregulated in the leaves, whereas more genes re upregulated and downregulated in the roots (Taheri et al., 2022). The organ heterogeneity of multiple gene expression is difficult to study using traditional methods and transcriptomic technology has helped to overcome this difficulty. In addition to the aspects mentioned above, the effects of biological factors such as disease, abiotic factors such as nutritional deficiencies, and extreme temperatures on the sugarcane transcriptome have also been studied (Li et al., 2023).

Although the relationships between the genic expression and drought resistance of sugarcane, as well as some cultivar-related studies, have been reported, these studies are insufficient; understanding the mechanism of drought resistance requires further exploration because of the complexity of the sugarcane genome as well as its source, and the development of modern sugarcane varieties (Pereira-Santana et al., 2017; Liu et al., 2018). To gain a more comprehensive understanding of the drought tolerance mechanisms for different sugarcane varieties, differences in transcriptomes of two sugarcane cultivar GT31, with weak drought tolerance, and F172 with strong drought tolerance were investigated in this study. Based on the transcriptome data, we further explored the differences in metabolic pathways and related gene expression between the different varieties under drought stress and confirmed that drought-resistant sugarcane responds to drought stress by regulating metabolic pathways and related gene expression, particularly the photosynthesis pathway. These results enrich our understanding of the molecular mechanisms underlying drought resistance in plants and provide a basis for sugarcane breeding.

## Materials and methods

### Plant material and sampling

In this study, plant materials from two sugarcane *Saccharum* L. cultivars, F172 and Guitang 31 (GT31), with strong and weak drought tolerance, developed by the Taiwan Sugar Research Institute (Taiwan, China) and Sugarcane Research Institute, Guangxi Academy of Agricultural Sciences (Nanning China), respectively, were used (Yang and Li, 1992; Li et al., 2011).

**Figure 1:**
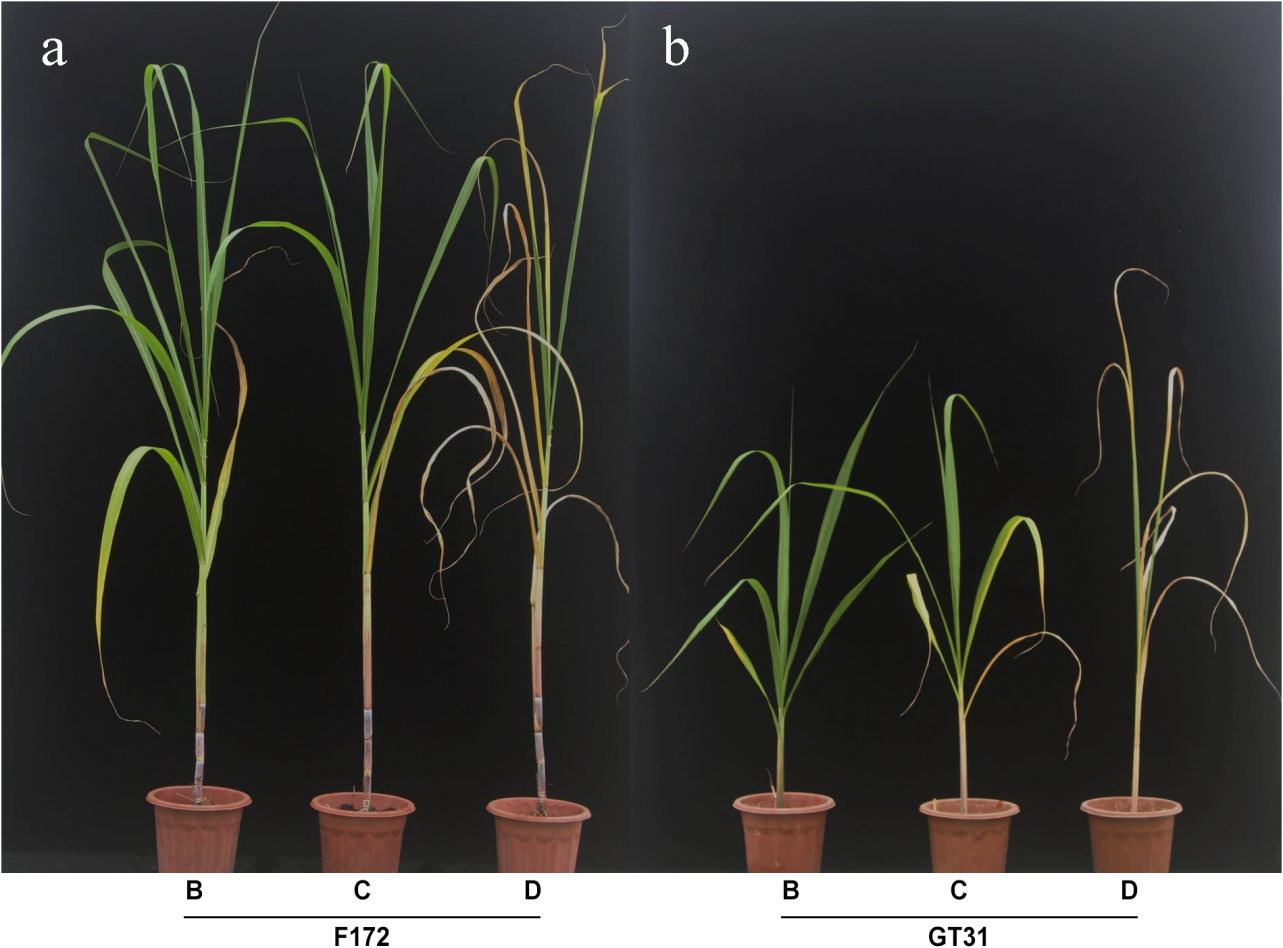
Appearance of two sugarcane varieties in drought tolerance. **(A)** Appearance of cultivar F172 under mild (left), moderate (middle), and severe (right) drought stress, respectively. **(B)** Appearance of cultivar GT31 under mild (left), moderate (middle), and severe (right) drought stress, respectively.

The sugarcane seedlings were observed and photographed at 4, 5, and 7 d after water withdrawal and were categorized under different drought treatment conditions: mild drought stress (B), moderate drought stress (C), and severe drought stress (D) (**Figure 1**). Both varieties showed significant changes across the three time points, and cultivar F172 showed stronger drought tolerance than Guitang31 (**Figure 1**). Sugarcane leaf tissues from cultivars F172 and GT31 were obtained under B, C, and K drought conditions for subsequent transcriptomic sequencing and photosynthetic rate detection. In parallel, leaf tissues from the two cultivars under normal watering conditions were collected at the same time points as controls for subsequent transcriptomic sequencing and detection of the photosynthetic rate, which are abbreviated as BCK (on day 4), CCK (on day 5), and DCK (on day 7).

The experiment was performed in triplicates for each condition and cultivar. All the leaf samples were flash-frozen with liquid nitrogen and stored at −80°C until further use.

### RNA extraction and RNA-seq analysis

RNA was extracted using the RNeasy Mini Kit (Qiagen, Beijing, China), followed by purification, fragmentation, and quality control using a NanoDrop 2000 (Thermo Scientific) and an Agilent 2100 Bioanalyzer (Agilent Technologies). Strand-specific libraries were obtained using dUTP for second-strand synthesis and subsequently sequenced on a BGISEQ-500 instrument (BGI, Shenzhen, China). The experiments were conducted in triplicates for each cultivar at each time point under drought (B, C, and K) and control (BCK, CCK, and DCK) conditions.

Preprocessing of the paired-end reads was performed using FASTP (v 0.23.1), and mapped to the sugarcane genome (NCBI accession: ASM2245720v1) using HISAT2 (v 2.20) (Kim et al., 2019). Raw read counts were quantified using the featureCounts software (SUBREAD v2.0.0) (Liao et al., 2014). Differential expression analysis was performed using the DESeq2 package (v1.38.3) (Love et al., 2014) for transcriptome comparisons between cultivars under the same conditions (GT31-B-vs-F172-B, GT31-C-vs-F172-C, GT31-D-vs-F172-D, GT31-BCK-vs-F172-BCK, GT31-CCK-vs-F172-CCK, and GT31-DCK-vs-F172-DCK) and between treatments and the corresponding controls for each cultivar (GT31-BCK-vs-GT31-B, F172-BCK-vs-F172-B, GT31-CCK-vs-GT31-C, F172-CCK-vs-F172-C, GT31-DCK-vs-GT31-D, and F172-DCK-vs-F172-D) to identify the differentially expressed genes (DEGs) with an absolute value of log2 FC > 1.0 and false discovery rate < 0.05. KEGG pathway analyses of the identified DEGs were conducted using ClusterProfiler (v4.3.1) (Yu et al., 2012).

### Temporal analysis

Clusters of genes with the same expression profile over different time points were identified using the short time-series expression miner (v1.3.13) (Ernst and Bar-Joseph, 2006) for cultivar F172 under drought stress (F172), cultivar F172 controls (F172CK), cultivar GT31 under drought stress (GT31), and cultivar GT31 controls (GT31CK). The statistical significance of the number of genes for each profile compared to the expected number was computed using a permutation-based test. Unique and common trend genes for significant profile clusters (*p* < 0.05) from the above-mentioned four groups were selected and KEGG enrichment analysis was performed as described above.

### Weighted correlation network analysis

Weighted correlation network analysis (v 1.69) (Langfelder and Horvath, 2008) was used to infer the network modules (parameters: softPower = 20, mergeCutHeight = 0.7, minModuleSize = 30)for 58,873 genes after filtering those with low expression levels (Fragments per kilobase of transcripts per million fragments mapped < 0.5). Module-trait associations were estimated using the correlation between module eigengenes and traits, including cultivars, drought conditions, and the strength of drought stress. Module–trait associations were considered statistically significant at *p* < 0.05. Trait-related genes with significant correlations were extracted from the module and subjected to KEGG enrichment analysis as described above. Hub genes in the modules were identified using the CytoHubba module in Cytoscape software.

### Measurement of photosynthetic rate

A portable photosynthesissystem (Li-6800, Li-COR Biosciences, Lincoln, NE, USA) was used to observe the net photosynthetic rate for the functional topvisible dewlap leaf (leaf + 1) of sugarcane. as previously described (Verma et al., 2020). The photosynthesis rate was measured with three biological replicates for each cultivar at each time point under both drought treatment and control conditions.

### Statistical analysis

The pheatmap package (v1.0.12) (https://CRAN.R-project.org/package=pheatmap) was used to plot the heatmaps. Differences were calculated using the t-test and were considered statistically significant at *p* < 0.05. Photosynthetic rate data were statistically analyzed using one-way ANOVA followed by Duncan’s multiple range test.

## Results

### Transcriptome analysis of the sugarcane cultivars

**Figure 2:**
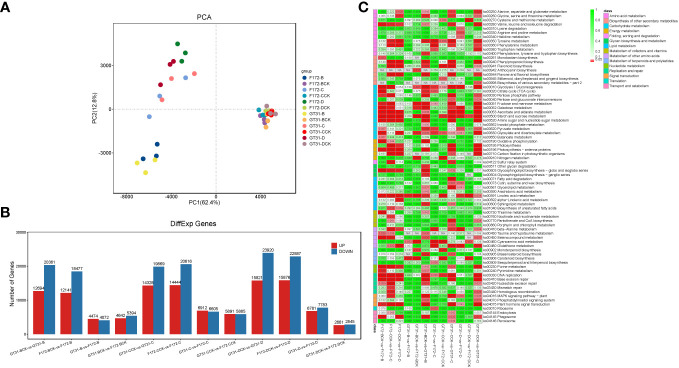
Transcriptome analysis of the sugarcane cultivars. **(A)** Principal component analysis (PCA) of transcriptome data. **(B)** Bar plot of the differentially expressed genes (DEGs) between cultivars in the same condition and between treatments and the corresponding controls for each cultivar, respectively. **(C)** Heatmap of relative enrichment qvalue of each pathway (rows) for each comparison (columns).

RNA sequencing generated a total of 379.6 G of raw data for all 36 samples (each in the range of 7.8 G–13.9 G) (**Table S1**). Approximately 373.8 G clean reads were obtained and passed through quality control; all samples were of high quality (Q20 ≥ 96.68% and Q30 ≥ 91.86%) (**Table S1**). The mapping rate of clean reads to the sugarcane reference genome ranged from 76.16% to 79.84% (**Table S1**). Principal component analysis showed low inter-replicate variability, and the samples in each group clustered together (**Figure 2A**). The principal component analysis clearly distinguished between the water deficit and control conditions, and the cultivars were also well separated (**Figure 2A**), indicating a large variability between the F172 and GT31 cultivars.

Differential gene expression analysis was performed between both cultivars grown under the same conditions, and between those grown under different conditions along with the corresponding controls (**Figure 2B**; **Table S2**). A total of 8,546, 10,036, 13,517, 11,776, 14,564, and 5,506 DEGs were identified between the GT31 and F172 cultivars under the same drought conditions, including B, BCK, C, CCK, D, and DCK, respectively (**Figure 2B**). On comparing intra-cultivar drought treatments and controls, we identified 33,058, 34,197, and 39,741 DEGs from the comparisons of three conditions (B vs. B-vs-BCK, C vs. C-vs-CCK, and D-vs-DCK) for strain GT31, and 30,618, 35,060, and 38,763 DEGs for strain F172, respectively (**Figure 2B**). In general, the number of DEGs between the intra-cultivar drought treatments and controls was much greater than that between the inter-cultivar differences under the same conditions. These DEGs between cultivars grown under the same conditions were mainly enriched in starch and sucrose metabolism, linoleic acid metabolism, glycolysis/gluconeogenesis, glyoxylate and dicarboxylate metabolism, nitrogen metabolism, carotenoid biosynthesis, and ribosome pathways among others (**Figure 2C**). Compared with the DEGs between cultivars under normal watering conditions, those under drought conditions enriched in pyruvate metabolism, beta-alanine metabolism, glutathione metabolism specifically under moderate and severe drough stress (**Figure 2C**). In the comparison groups BCK-vs-GT31-B, CCK-vs-GT31-C, and DCK-vs-GT31-D, we identified 33,058 DEGs (12,697 up and 20,361 down), 34,197 DEGs (14,328 up and 19,869 down), and 39,741 DEGs (15,821 up and 23,920 down) for strain GT31, and 30,618 DEGs (12,141 up and 18,477 down), 35,060 DEGs (14,444 up and 20,616 down), and 38,763 DEGs (15,876 up and 22,887 down) for stain F172, respectively (**Figure 2B**). The DEGs between cultivars grown under different conditions along with the corresponding controls were mainly enriched in starch and sucrose metabolism, linoleic acid metabolism, glycolysis/gluconeogenesis, glyoxylate and dicarboxylate metabolism, valine, leucine and isoleucine degradation, phenylpropanoid biosynthesis, flavone and flavonol biosynthesis, photosynthesis-antenna proteins, glycosphingolipid biosynthesis (globo- and isoglobo- series), and phagosome pathways among others (**Figure 2C**). Many DEGs were identified between the drought stress and control groups for the same time points for both cultivars (**Figure 2B**; **Table S2**), indicating that drought stress has a significant impact on gene expression.

### Comparison of trends between F172 and GT31 cultivars under in different drought stress conditions

**Figure 3:**
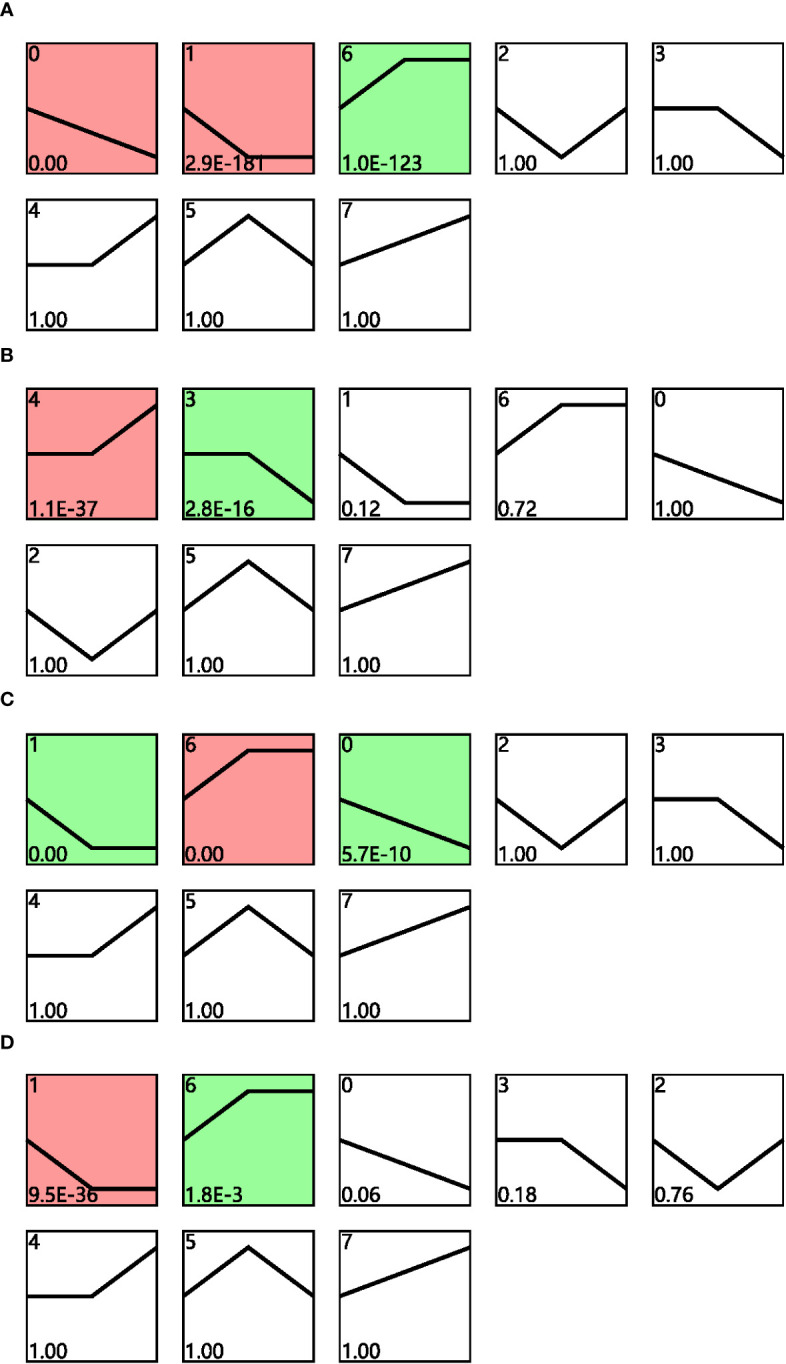
STEM analysis of gene expression profiles. Distinct expression profiles of cultivar F172 in drought stress **(A)**, cultivar F172 controls **(B)**, cultivar GT31 in drought stress **(C)**, and cultivar GT31 controls **(D)** were identified, respectively. The profiles were ordered by pvalue and those highlighted with colored background were significant profiles.

Time-series expression analysis was performed to examine the dynamic transcriptomic differences in drought tolerance between cultivars. The gene expression patterns for cultivars grown under different conditions and their corresponding controls were analyzed for both cultivars across different drought time points, and the profiles with *p* < 0.05 in the permutation test were considered as significant (**Figure 3**). Profiles 0, 1, and 6 were considered to be significant for cultivar F172 in drought stress (**Figure 3A**; **Table S3**); profiles 4 and 3 were considered to be significant for cultivar F172 controls (**Figure 3B**; **Table S4**); profiles 1, 6, and 0 were considered to be significant for cultivar GT31 under drought stress (**Figure 3C**; **Table S5**); and profiles 1 and 6 were considered to be significant for cultivar GT31 controls (**Figure 3D**; **Table S6**).

**Figure 4:**
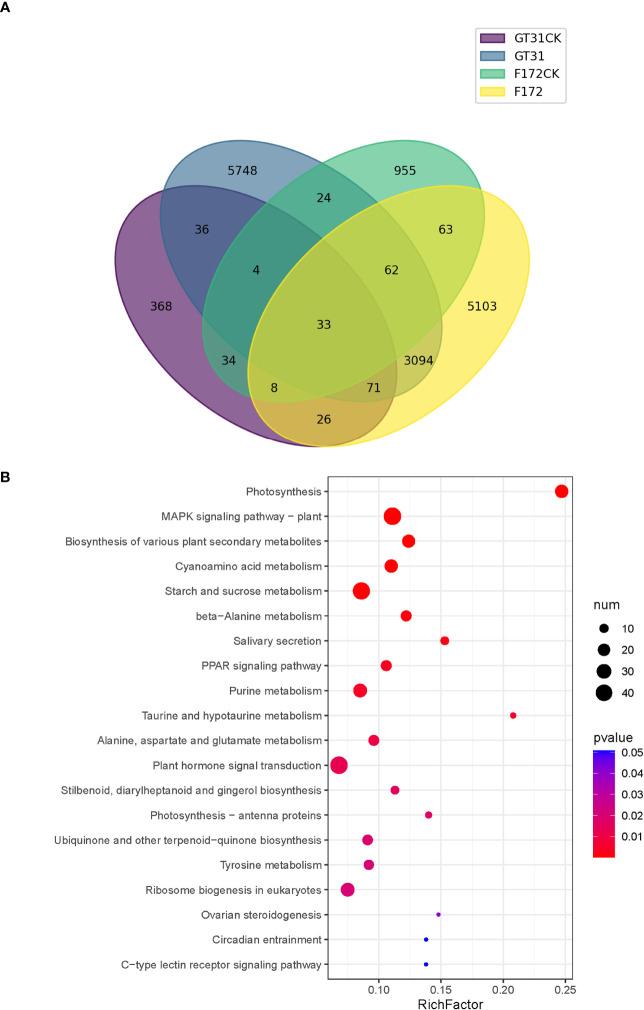
Comparison of trends between F172 and GT31 in different drought stress. **(A)** identification of unique trend genes in cultivar F172 in drought stress (F172) compared with cultivar F172 controls (F172CK), cultivar GT31 in drought stress (GT31), and cultivar GT31 controls (GT31CK). **(B)** Significantly enriched KEGG pathways of the unique trend genes in F172.

To identify the trend genes specific to F172 in response to drought stress, an intersection analysis was conducted for the trend genes in different groups, including the cultivar F172 under drought stress (F172), cultivar F172 controls (F172CK), GT31 under drought stress (GT31), and GT31 controls (GT31CK) (**Figure 4A**). Among them, 5,103 genes exhibited a specific trend in the F172 group that was inconsistent with those in the GT31 and the F172 controls, potentially related to the drought tolerance of F172. The KEGG pathway enrichment results showed that these genes were significantly enriched in photosynthesis, mitogen-activated protein kinases (MAPK) signaling pathway, biosynthesis of various plant secondary metabolites, and cyanoamino acid metabolism pathways (**Figure 4B**).

### Weighted gene co-expression network analysis of F172 and GT31 cultivars

**Figure 5:**
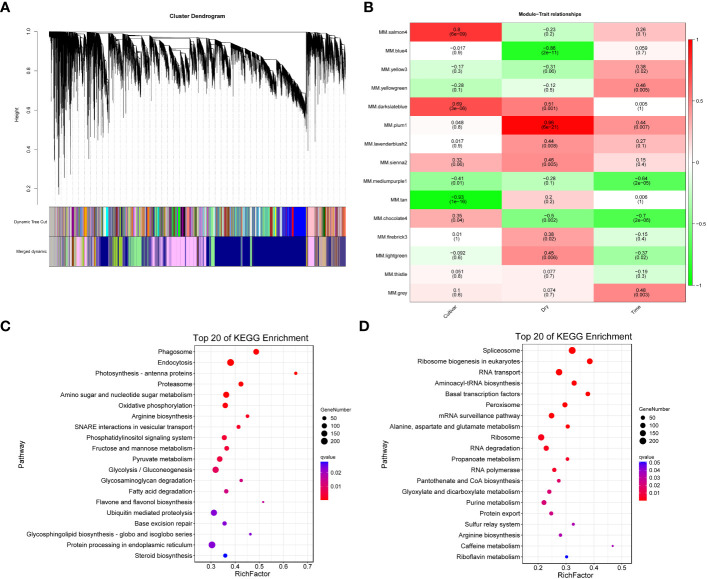
Weighted Gene Co-expression Network Analysis (WGCNA). **(A)** WGCNA module detection. **(B)** Heatmap of module - trait correlation, displaying the correlation values for each module (rows) and each trait (columns). **(C)** Significantly enriched KEGG pathways of the genes in blue4 module. **(D)** Significantly enriched KEGG pathways of the genes in plum1 module.

We employed WGCNA to identify potential co-expression modules and key regulatory networks, and further elucidate their roles in the response of the F172 cultivar to drought stress. WGCNA categorized all genes into 15 modules (**Figure 5A**). Module-trait relationships were explored to extract significant associations between cultivars, drought conditions, strength of drought stress, and modules (**Figure 5B**). The blue4 and plum1 modules showed significant negative (–0.86, p < 0.01) and positive (0.96, p < 0.01) correlations, respectively, with drought conditions, (**Figure 5B**). The tan and salmon4 modules showed significant negative (-0.93, p < 0.01) and positive (0.8, p < 0.01) correlations, respectively, with the cultivars (**Figure 5B**).

All unique trend genes (**Figure 4A**) were mapped to weighted correlation network analysis modules (**Table S7**). Among the unique trend genes identified in F172 compared to GT31 and controls, most were in the blue4 (1,301) and plum1 (983) modules (**Table S7**); these were then used for KEGG enrichment analysis. Genes in the blue4 module were mainly enriched in phagosome, endocytosis, photosynthesis-antenna proteins, amino sugar and nucleotide sugar metabolism, and oxidative phosphorylation, among others (**Figure 5C**; **Table S8**). Those genes in the plum1 module were mainly enriched in spliceosome, RNA transport, aminoacyl-tRNA biosynthesis, basal transcription factors, peroxisome, and alanine, aspartate, and glutamate metabolism, among others (**Figure 5D**). Several enriched pathways, including those of photosynthesis, purine metabolism, starch and sucrose metabolism, beta-alanine metabolism, photosynthesis-antenna proteins, and plant hormone signal transduction, were shared between those genes in the blue4 module and the unique trend genes in F172 (**Figure 5C**; **Table S8**). The hub genes identified in the blue4 module included *ARF14* (auxin response factor 14) and *Os10g0147400* (similar to auxin influx carrier protein), which are associated with the plant hormone signal transduction pathway, and *Os02g0733300* (glycoside hydrolase gene), which is associated with the starch and sucrose metabolism pathway (**Table S9**). The hub genes of plum1 module included the spliceosome-related genes *RS31* (serine/arginine-rich splicing factor RS31) and *CLPC1* (chaperone protein ClpC1, chloroplastic), and peroxisome-related genes *PEX1* (peroxisomal biogenesis factor 1) and *PEX2* (peroxisomal biogenesis factor 2) (**Table S10**).

### Differences in the photosynthesis pathway between F172 and GT31 cultivars

**Figure 6:**
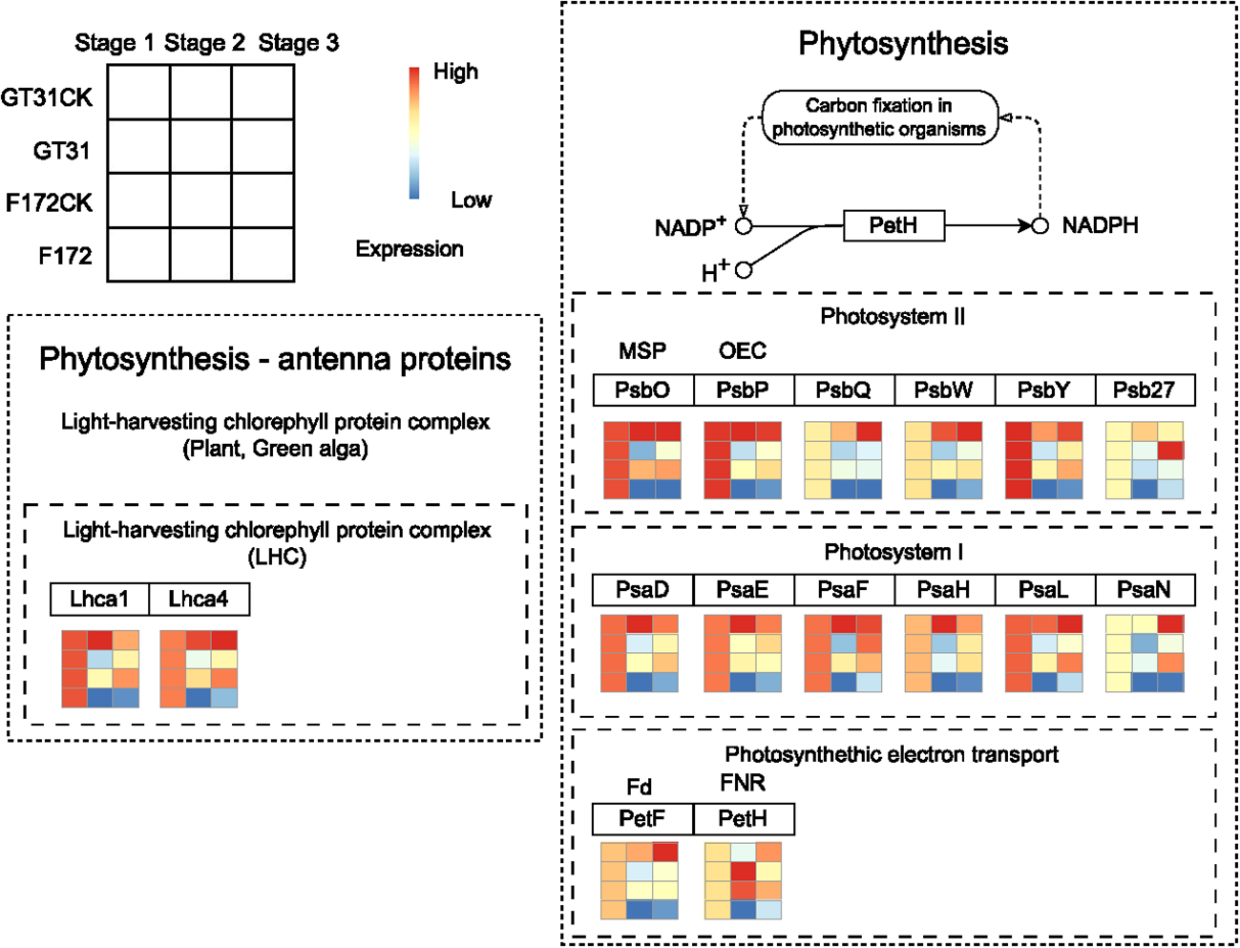
The expression pattern of genes in blue4 module which involved in Phytosynthesis - antenna proteins and Phytosynthesis pathways for cultivar F172 in drought stress (F172), cultivar F172 controls (F172CK), cultivar GT31 in drought stress (GT31), and cultivar GT31 controls (GT31CK), respectively.

Photosynthesis is closely associated with drought stress in plants (**Figure 6**). In the photosynthesis-antenna protein pathway for F172, the expression levels of *Lhca1* (light-harvesting complex I chlorophyll a/b binding protein 1) and *Lhca4* (light-harvesting complex I chlorophyll a/b binding protein 4) were high at stage 1 and significantly decreased at later stages, exhibiting a distinct downward trend (**Figure 6**). Further, *PsbO* (photosystem II oxygen-evolving enhancer protein 1), *PsbP* (photosystem II oxygen-evolving enhancer protein 2), *PsbQ* (photosystem II oxygen-evolving enhancer protein 3), *PsbW* (photosystem II PsbW protein), *PsbY* (photosystem II PsbY protein), *Psb27* (photosystem II Psb27 protein), *PsaD* (photosystem I subunit II), *PsaE* (photosystem I subunit IV), *PsaF* (photosystem I subunit III), *PsaH* (photosystem I subunit VI), *PsaL* (photosystem I subunit XI), *PsaN* (photosystem I subunit PsaN), *PetF* (ferredoxin), and *petH* (ferredoxin–NADP+ reductase), which participate the photosynthesis pathway, also showed a similar expression pattern for F172 (**Figure 6**).

**Figure 7:**
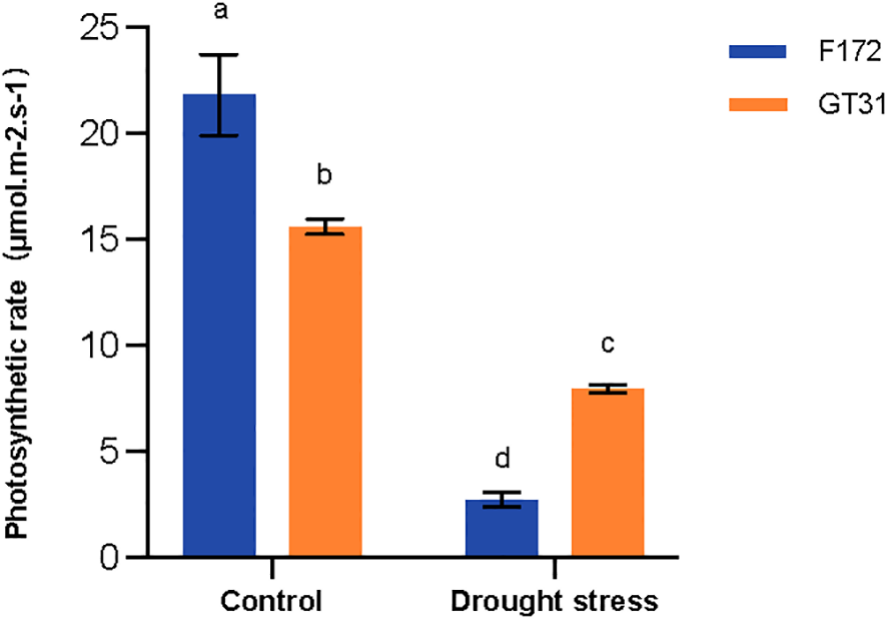
Photosynthetic rate of cultivars F172 and GT31 in drought stress and the corresponding controls. Different letters indicate significant differences (Duncan’s test at P < 0.05).

The photosynthetic rates for two cultivars F172 and GT31 were significantly down-regulated under drought conditions. However, this decrease was more pronounced for the F172 variety, which was consistent with the declining trend observed in the expression of genes related to the photosynthetic pathway as described above (**Figure 7**). These results indicate that drought stress resulted in a significantly greater reduction in photosynthesis for cultivar F172, which is associated with stronger drought resistance.

## Discussion

Sugarcane is an important commercial crop of global significance, more than 80% of the world’s sugar production is derived from sugarcane, which is grown in more than 90 nations (Barnabas et al., 2015). Drought limits sugarcane production (Liu et al., 2021), and different sugarcane cultivars react differently to drought stress (da Silva et al., 2017). In this study, principal component analysis could discriminate between two different sugarcane cultivars under water deficit conditions, but not when the moisture level was normal (**Figure 2A**). Drought can also alter plant gene expression (Takahashi et al., 2018; Bashir et al., 2019). In this study, based on the DEGs between F172 and GT31 under drought stress, the results of the KEGG pathway analysis showed that starch and sucrose metabolism, and glycolysis/gluconeogenesis were extensively enriched for all comparisons (**Figures 2B, C**). Changes in these two metabolic pathways may be some of the most common responses to drought because these pathways are also significantly enriched for the IACSP97-7065, IACSP94-2094, and ROC22 sugarcane varieties under drought stress (Contiliani et al., 2022; Yang et al., 2022).

Many other physiological and metabolic processes in plants are involved in the drought stress response; Takahashi et al. (2018) reported that drought inhibits photosynthesis; further, MAPK cascades modulate plant tolerance to drought (Bashir et al., 2019). Drought can also affect the levels of secondary plant metabolites such as calcium, anthocyanins, flavonoids, phenolic acids, chlorophyll and saponins (Yu et al., 2020; Contiliani et al., 2022; Yang et al., 2022). Wan et al. (2021) found that changes in the cyanoamino acid metabolism pathway might be a key factor causing the difference in drought resistance between two cherry rootstocks. In this study, based on the unique trend of genes in F172 compared to that of GT31 and the controls, the results of the KEGG pathway analysis indicated that photosynthesis, MAPK signaling pathway, biosynthesis of various plant secondary metabolites, and cyanoamino acid metabolism pathways were significantly enriched in F172. In addition to these pathways, other enriched metabolic pathways, such as cyanoamino acid, starch, sucrose, urine, and ribosome biogenesis in eukaryotes (**Figure 4B**), have rarely been associated with drought tolerance in plants. The role of genes exhibiting specific trends in these pathways in the F172 group deserves further investigation.

Weighted correlation network analysis has been an important method used in previous studies on plant drought resistance (Yu et al., 2020; Cao et al., 2021). In *Arabidopsis thaliana*, both blue and salmon modules responded to drought (Sharma et al., 2018). Moreover, the blue module cprresponded to drought resistance traits in sunflower and Tartary buckwheat (Meng et al., 2022; Wu et al., 2022). In this study, the results of the weighted correlation network analysis demonstrated that the blue4 module was significantly negatively correlated with drought conditions, and the salmon4 module was significantly positively correlated with the cultivars (**Figure 5B**). In addition to the aforementioned metabolic pathways, several other pathways are involved in plant drought resistance, such as the plant hormone signal transduction pathways (Verma et al., 2016). The purine and phenylpropanoid metabolism pathways in *Dendrobium sinense* and Arabidopsis are also involved in the drought stress response (Watanabe et al., 2010; Zhang et al., 2021). Metabolome analysis showed that drought stress resulted in an increase in β-alanine in tomato fruit (Asakura et al., 2021). In addition, transcriptome analysis suggested that the photosynthesis-antenna protein pathway in Shanlan upland rice and peanuts is involved in the drought stress response (Ren et al., 2021; Zhou et al., 2022). In this study, KEGG pathway analysis showed that genes with unique trends in the blue module were enriched in photosynthesis, purine metabolism, starch and sucrose metabolism, alanine metabolism, photosynthesis-antenna proteins, and plant hormone signal transduction pathways (**Figure 5C**).

The most prominent effects of drought on crops are related to the germination and photosynthesis processes (Flexas et al., 2004; Nadeem et al., 2019). In the present study, both the photosynthesis-antenna protein and photosynthesis pathways were enriched for the drought-resistant variety, F172, under drought stress (**Figures 4B**, **5C**). The proteins involved in the two pathways increased at normal moisture levels, but decreased under water-deficit conditions (**Figure 6**). These results indicated that under water-deficit conditions, drought-resistant sugarcane varieties might mitigate the effects of drought by adjusting the photosynthesis process. Reduced photosynthesis can save plenty of water for the plant itself, which may be the basis of drought resistance oin the F172 variety of sugarcane (**Figure 7**).

## Conclusion

In the present study, we used comparative temporal analysis to unveil the differences in gene expression trends during drought stress between two sugarcane cultivars with differing drought tolerance levels. By performing WGCNA, we found that the two cultivars showed different trends in genes related to photosynthesis, MAPK signaling, biosynthesis of various plant secondary metabolites, and cyanoamino acid metabolism pathways in response to drought stress. The most notable change in this process was the reduction in expression of genes related to photosynthesis; the corresponding decrease in photosynthesis may be an important strategy adopted by plants to cope with drought. The bioinformatic analysis strategies used in this study may also be valuable for uncovering key factors related to important traits by comparing different cultivars.

## Data availability statement

The original contributions presented in the study are publicly available. This data can be found here: https://www.ncbi.nlm.nih.gov/bioproject/PRJNA975299.

## Author contributions

HL, RY and RZ: conceptualization. HL, XL and HZ: validation. KZ and JW: data curation. RZ, YG and HL: writing original draft preparation. LT, HZ and XL. writing review and editing. All authors contributed to the article and approved the submitted version.

## References

[B1] Agurla S. Gahir S. Munemasa S. Murata Y. Raghavendra A. S. (2018). Mechanism of stomatal closure in plants exposed to drought and cold stress. Adv. Exp. Med. Biol. 1081, 215–232. doi: 10.1007/978-981-13-1244-1_12 30288712

[B2] Asakura H. Yamakawa T. Tamura T. Ueda R. Taira S. Saito Y.. (2021). Transcriptomic and Metabolomic Analyses Provide Insights into the Upregulation of Fatty Acid and Phospholipid Metabolism in Tomato Fruit under Drought Stress. J. Agric. Food Chem. 69, 2894–2905. doi: 10.1021/acs.jafc.0c06168 33645220

[B3] Barnabas L. Ramadass A. Amalraj R. S. Palaniyandi M. Rasappa V. (2015). Sugarcane proteomics: An update on current status, challenges, and future prospects. Proteomics 15, 1658–1670. doi: 10.1002/pmic.201400463 25641866

[B4] Bashir K. Matsui A. Rasheed S. Seki M. (2019). Recent advances in the characterization of plant transcriptomes in response to drought, salinity, heat, and cold stress. F1000Res 8, 658. doi: 10.12688/f1000research.18424.1 PMC6518435 31131087

[B5] Belesini A. A. Carvalho F. M. S. Telles B. R. de Castro G. M. Giachetto P. F. Vantini J. S.. (2017). *De novo* transcriptome assembly of sugarcane leaves submitted to prolonged water-deficit stress. Genet. Mol. Res. 16, gmr16028845. doi: 10.4238/gmr16028845 28549198

[B6] Cao L. Lu X. Wang G. Zhang P. Fu J. Wang Z.. (2021). Transcriptional regulatory networks in response to drought stress and rewatering in maize (*Zea mays* L.). Mol. Genet. Genomics 296, 1203–1219. doi: 10.1007/s00438-021-01820-y 34601650

[B7] Chen Y. Li Z. Sun T. Wang D. Wang Z. Zhang C.. (2022). Sugarcane scDREB2B-1 confers drought stress tolerance in transgenic nicotiana benthamiana by regulating the ABA signal, ROS level and stress-related gene expression. Int. J. Mol. Sci. 23, 9557. doi: 10.3390/ijms23179557 36076957 PMC9455921

[B8] Conti V. Parrotta L. Romi M. Del Duca S. Cai G. (2023). Tomato biodiversity and drought tolerance: A multilevel review. Int. J. Mol. Sci. 24, 10044. doi: 10.3390/ijms241210044 37373193 PMC10298849

[B9] Contiliani D. F. de Oliveira Nebó J. F. C. Ribeiro R. V. Andrade L. M. Peixoto Júnior R. F. Lembke C. G.. (2022). Leaf transcriptome profiling of contrasting sugarcane genotypes for drought tolerance under field conditions. Sci. Rep. 12, 9153. doi: 10.1038/s41598-022-13158-5 35650424 PMC9160059

[B10] da Silva M. D. de Oliveira Silva R. L. Ferreira Neto J. R. C. Benko-Iseppon A. M. Kido E. A. (2017). Genotype-dependent regulation of drought-responsive genes in tolerant and sensitive sugarcane cultivars. Gene 633, 17–27. doi: 10.1016/j.gene.2017.08.022 28855118

[B11] Dinesh Babu K. S. Janakiraman V. Palaniswamy H. Kasirajan L. Gomathi R. Ramkumar T. R. (2022). A short review on sugarcane: its domestication, molecular manipulations and future perspectives. Genet. Resour. Crop Evol. 69, 2623–2643. doi: 10.1007/s10722-022-01430-6 36159774 PMC9483297

[B12] Ernst J. Bar-Joseph Z. (2006). STEM: a tool for the analysis of short time series gene expression data. BMC Bioinf. 7, 191. doi: 10.1186/1471-2105-7-191 PMC1456994 16597342

[B13] Ferreira T. H. S. Tsunada M. S. Bassi D. Araújo P. Mattiello L. Guidelli G. V.. (2017). Sugarcane water stress tolerance mechanisms and its implications on developing biotechnology solutions. Front. Plant Sci. 8. doi: 10.3389/fpls.2017.01077 PMC5481406 28690620

[B14] Flexas J. Bota J. Loreto F. Cornic G. Sharkey T. D. (2004). Diffusive and metabolic limitations to photosynthesis under drought and salinity in C(3) plants. Plant Biol. (Stuttg). 6, 269–279. doi: 10.1055/s-2004-820867 15143435

[B15] Gentile A. Dias L. I. Mattos R. S. Ferreira T. H. Menossi M. (2015). MicroRNAs and drought responses in sugarcane. Front. Plant Sci. 6. doi: 10.3389/fpls.2015.00058 PMC4337329 25755657

[B16] Gentile A. Ferreira T. H. Mattos R. S. Dias L. I. Hoshino A. A. Carneiro M. S.. (2013). Effects of drought on the microtranscriptome of field-grown sugarcane plants. Planta 237, 783–798. doi: 10.1007/s00425-012-1795-7 23129215 PMC3579473

[B17] Kim D. Paggi J. M. Park C. Bennett C. Salzberg S. L. (2019). Graph-based genome alignment and. genotyping with HISAT2 and HISAT-genotype. Nat. Biotechnol. 37, 907–915. doi: 10.1038/s41587-019-0201-4 31375807 PMC7605509

[B18] Langfelder P. Horvath S. (2008). WGCNA: an R package for weighted correlation network analysis. BMC Bioinf. 9, 559. doi: 10.1186/1471-2105-9-559 PMC2631488 19114008

[B19] Li A. M. Liao F. Wang M. Chen Z. L. Qin C. X. Huang R. Q.. (2023). Transcriptomic and proteomic landscape of sugarcane response to biotic and abiotic stressors. Int. J. Mol. Sci. 24, 8913. doi: 10.3390/ijms24108913 37240257 PMC10219567

[B20] Li X. Liu Z. Zhao H. Deng X. Su Y. Li R.. (2022). Overexpression of sugarcane scDIR genes enhances drought tolerance in nicotiana benthamiana. Int. J. Mol. Sci. 23, 5340. doi: 10.3390/ijms23105340 35628151 PMC9141896

[B21] Li H. G. Tan Y. M. Tan F. Wang L. W. Yang R. Z. Liu X. J. (2011). Breeding of new sugarcane variety guitang 31 with high productivity and sugar content and ratoon ability. Seed 30 (08), 116–118. doi: 10.16590/j.cnki.1001-4705.2011.08.061

[B22] Liao Y. Smyth G. K. Shi W. (2014). featureCounts: An efficient general purpose program for assigning sequence reads to genomic features. Bioinformatics 30, 923–930. doi: 10.1093/bioinformatics/btt656 24227677

[B23] Liu X. Zhang R. Ou H. Gui Y. Wei J. Zhou H.. (2018). Comprehensive transcriptome analysis reveals genes in response to water deficit in the leaves of *Saccharum narenga* (Nees ex Steud.) hack. BMC Plant Biol. 18, 250. doi: 10.1186/s12870-018-1428-9 30342477 PMC6195978

[B24] Liu Q. Zhao X. Liu Y. Xie S. Xing Y. Dao J.. (2021). Response of sugarcane rhizosphere bacterial community to drought stress. Front Microbiol. 12. doi: 10.3389/fmicb.2021.716196 PMC8527094 34690961

[B25] Love M. I. Huber W. Anders S. (2014). Moderated estimation of fold change and dispersion for RNA-seq data with DESeq2. Genome Biol. 15, 550. doi: 10.1186/s13059-014-0550-8 25516281 PMC4302049

[B26] Meena M. R. Kumar R. Chinnaswamy A. Karuppaiyan R. Kulshreshtha N. Ram B. (2020). Current breeding and genomic approaches to enhance the cane and sugar productivity under abiotic stress conditions. 3 Biotech. 10, 440. doi: 10.1007/s13205-020-02416-w PMC7501393 33014683

[B27] Meng H. L. Sun P. Y. Wang J. R. Sun X. Q. Zheng C. Z. Fan T.. (2022). Comparative physiological, transcriptomic, and WGCNA analyses reveal the key genes and regulatory pathways associated with drought tolerance in Tartary buckwheat. Front. Plant Sci. 13. doi: 10.3389/fpls.2022.985088 PMC9575659 36262653

[B28] Nadeem M. Li J. Yahya M. Sher A. Ma C. Wang X.. (2019). Research progress and perspective on drought stress in legumes: A review. Int. J. Mol. Sci. 20 (10), 2541. doi: 10.3390/ijms20102541 31126133 PMC6567229

[B29] Nawae W. Shearman J. R. Tangphatsornruang S. Punpee P. Yoocha T. Sangsrakru D.. (2020). Differential expression between drought-tolerant and drought-sensitive sugarcane under mild and moderate water stress as revealed by a comparative analysis of leaf transcriptome. PeerJ 8, e9608. doi: 10.7717/peerj.9608 33240580 PMC7676377

[B30] Ozturk M. Turkyilmaz Unal B. García-Caparrós P. Khursheed A. Gul A. Hasanuzzaman M. (2021). Osmoregulation and its actions during the drought stress in plants. Physiol. Plant 172, 1321–1335. doi: 10.1111/ppl.13297 33280137

[B31] Pereira-Santana A. Alvarado-Robledo E. J. Zamora-Briseño J. A. Ayala-Sumuano J. T. Gonzalez-Mendoza V. M. Espadas-Gil F.. (2017). Transcriptional profiling of sugarcane leaves and roots under progressive osmotic stress reveals a regulated coordination of gene expression in a spatiotemporal manner. PLoS One 12, e0189271. doi: 10.1371/journal.pone.0189271 29228055 PMC5724895

[B32] Ren J. Zhang H. Shi X. Ai X. Dong J. Zhao X.. (2021). Genome-wide identification of key candidate microRNAs and target genes associated with peanut drought tolerance. DNA Cell Biol. 40, 373–383. doi: 10.1089/dna.2020.6245 33373540

[B33] Selvi A. Devi K. Manimekalai R. Prathima P. T. (2020). Comparative analysis of drought-responsive transcriptomes of sugarcane genotypes with differential tolerance to drought. 3 Biotech. 10, 236. doi: 10.1007/s13205-020-02226-0 PMC7203378 32399386

[B34] Sharma R. Singh G. Bhattacharya S. Singh A. (2018). Comparative transcriptome meta-analysis of Arabidopsis thaliana under drought and cold stress. PLoS One 13, e0203266. doi: 10.1371/journal.pone.0203266 30192796 PMC6128483

[B35] Taheri S. Gantait S. Azizi P. Mazumdar P. (2022). Drought tolerance improvement in Solanum lycopersicum: an insight into "OMICS" approaches and genome editing. 3 Biotech. 12, 63. doi: 10.1007/s13205-022-03132-3 PMC8825918 35186660

[B36] Takahashi F. Kuromori T. Sato H. Shinozaki K. (2018). Regulatory gene networks in drought stress responses and resistance in plants. Adv Exp Med Biol. 1081, 189–214. doi: 10.1007/978-981-13-1244-1_11 30288711

[B37] Tardieu F. Simonneau T. Muller B. (2018). The physiological basis of drought tolerance in crop plants: A scenario-dependent probabilistic approach. Annu. Rev. Plant Biol. 69, 733–759. doi: 10.1111/ppl.13297 29553801

[B38] Verma V. Ravindran P. Kumar P. P. (2016). Plant hormone-mediated regulation of stress responses. BMC Plant Biol. 16, 86. doi: 10.1186/s12870-016-0771-y 27079791 PMC4831116

[B39] Verma K. K. Song X. P. Zeng Y. Li D. M. Guo D. J. Rajput V. D.. (2020). Characteristics of leaf stomata and their relationship with photosynthesis in *Saccharum officinarum* under drought and silicon application. ACS Omega. 4, 5(37):24145–24153. doi: 10.1021/acsomega.0c03820 PMC7513552 32984737

[B40] Wan T. Feng Y. Liang C. Pan L. He L. Cai Y. (2021). Metabolomics and transcriptomics analyses of two contrasting cherry rootstocks in response to drought stress. Biology 10, 201. doi: 10.3390/biology10030201 33800812 PMC8001747

[B41] Watanabe S. Nakagawa A. Izumi S. Shimada H. Sakamoto A. (2010). RNA interference-mediated suppression of xanthine dehydrogenase reveals the role of purine metabolism in drought tolerance in Arabidopsis. FEBS Lett. 584, 1181–1186. doi: 10.1016/j.febslet.2010.02.023 20153325

[B42] Wu Y. Wang Y. Shi H. Hu H. Yi L. Hou J. (2022). Time-course transcriptome and WGCNA analysis revealed the drought response mechanism of two sunflower inbred lines. PLoS One 17, e0265447. doi: 10.1371/journal.pone.0265447 35363798 PMC8974994

[B43] Yang L. Li Y. (2022). Analysis of photosynthetic rate and sugar accumulation of Taitang 172. Guangxi Agric. Sci. 02, 61–62.

[B44] Yang S. Chu N. Zhou H. Li J. Feng N. Su J.. (2022). Integrated Analysis of Transcriptome and Metabolome Reveals the Regulation of Chitooligosaccharide on Drought Tolerance in Sugarcane (*Saccharum* spp. Hybrid) under Drought Stress. Int. J. Mol. Sci. 23, 9737. doi: 10.3390/ijms23179737 36077135 PMC9456405

[B45] Yu B. Liu J. Wu D. Liu Y. Cen W. Wang S.. (2020). Weighted gene coexpression network analysis-based identification of key modules and hub genes associated with drought sensitivity in rice. BMC Plant Biol. 20, 478. doi: 10.21203/rs.3.rs-56040/v2 33081724 PMC7576772

[B46] Yu G. Wang L. G. Han Y. He Q. Y. (2012). clusterProfiler: An R package for comparing biological themes among gene clusters. OMICS 16, 284–287. doi: 10.1089/omi.2011.0118 22455463 PMC3339379

[B47] Zhang C. Chen J. Huang W. Song X. Niu J. (2021). Transcriptomics and metabolomics reveal purine and phenylpropanoid metabolism response to drought stress in dendrobium sinense, an endemic orchid species in hainan island. Front. Genet. 12. doi: 10.3389/fgene.2021.692702 PMC8283770 34276795

[B48] Zhou S. He L. Lin W. Su Y. Liu Q. Qu M.. (2022). Integrative analysis of transcriptome and metabolism reveals potential roles of carbon fixation and photorespiratory metabolism in response to drought in Shanlan upland rice. BMC Genomics 23, 862. doi: 10.1186/s12864-022-09094-3 36585635 PMC9805275

